# Extracellular Vesicles in Bone Metastasis: Key Players in the Tumor Microenvironment and Promising Therapeutic Targets

**DOI:** 10.3390/ijms21186680

**Published:** 2020-09-12

**Authors:** Takaaki Tamura, Yusuke Yoshioka, Shinichi Sakamoto, Tomohiko Ichikawa, Takahiro Ochiya

**Affiliations:** 1 Department of Molecular and Cellular Medicine, Tokyo Medical University, 6-7-1 Nishishinjuku, Shinjuku-ku, Tokyo 160-0023, Japan; t-tamura@tokyo-med.ac.jp (T.T.); yyoshiok@tokyo-med.ac.jp (Y.Y.); 2 Department of Urology, Graduate School of Medicine, Chiba University, 1-8-1 Inohana, Chuo-ku, Chiba 260-8670, Japan; rbatbat1@gmail.com (S.S.); tomohiko_ichikawa@faculty.chiba-u.jp (T.I.)

**Keywords:** extracellular vesicles, bone metastasis, tumor microenvironment

## Abstract

Extracellular vesicles (EVs) are lipid membranous vesicles that are released from every type of cell. It has become clear that EVs are involved in a variety of biological phenomena, including cancer progression, and play critical roles in intracellular communication through the horizontal transfer of cellular cargoes such as proteins, DNA fragments, RNAs including mRNA and non-coding RNAs (microRNA, piRNA, and long non-coding RNA) and lipids. The most common cause of death associated with cancer is metastasis. Recent investigations have revealed that EVs are deeply associated with metastasis. Bone is a preferred site of metastasis, and bone metastasis is generally incurable and dramatically affects patient quality of life. Bone metastasis can cause devastating complications, including hypercalcemia, pathological fractures, spinal compression, and bone pain, which result in a poor prognosis. Although the mechanisms underlying bone metastasis have yet to be fully elucidated, increasing evidence suggests that EVs in the bone microenvironment significantly contribute to cancer progression and cancer bone tropism. Emerging evidence on EV functions in bone metastasis will facilitate the discovery of novel treatments. In this review, we will discuss the remarkable effects of EVs, especially on the tumor microenvironment in bone.

## 1. Introduction

The existence of membrane-enclosed structures found in the extracellular space has been known for more than 70 years; Chargaff and West first reported platelet-derived procoagulant particles after high-speed centrifugation of human plasma in 1946 [1]. In the 1960s, lipid-rich particles were observed by researchers describing coagulation- or calcification-inducing factors in plasma or bones, respectively [2,3]. In the 1970s and 1980s, multiple studies have demonstrated the presence of membrane-like vesicle structures in other various kinds of solid tissues, physiological fluids, and cell culture supernatants. Prostasomes, which are well known in urology and released from prostate epithelial cells in the human seminal fluid, were also reported for the first time in this era [4,5,6,7,8,9]. These vesicles were called a variety of names depending on their size and origin. However, their designations were vaguely defined, and their isolation methods were not well established. Therefore, the International Society for Extracellular Vesicles (ISEV), founded in 2012 in Sweden, recommends the use of extracellular vesicles (EVs) as a generic term for the vesicles secreted by these cells. The nomenclature of this vesicles is specified in the Minimal Information for Studies of Extracellular Vesicles (MISEV) 2018 which is guidelines proposed by ISEV. Three hundred and eighty-two expert authors in the field contributed to the MISEV guidelines, and 94% endorsed the nomenclature recommendation [10,11].

EVs are lipid membranous vesicles that are released from every type of cell. They are conventionally classified into three main categories: exosomes (approximately 100 nm), microvesicles (approximately 1 μm), and apoptotic bodies (greater than 1 μm) [12]. As previously mentioned, all three classes of EVs differ in not only size but also morphology, content, mode of generation, and mechanism of release. More specifically, exosomes are formed by the inward budding of early endosomes to form multivesicular bodies (MVBs). These MVBs fuse with the limiting plasma membrane to release exosomes into the extracellular space. Microvesicles originate by direct shedding or budding from the plasma membrane. Apoptotic bodies are released from the cell undergoing programmed cell death [13]. The term EVs is a collective term including many other names of vesicles; microparticle, ectosomes, and oncosomes, etc. It has been showed that each vesicle contains a slightly different group of molecules, which is due to the different biogenesis of each vesicle [10]. Furthermore, Théry et al. came up with a more streamlined nomenclature system of EVs. They defined vesicles <100 nm as small EVs, <200 nm as medium EVs, and >200 nm as large EVs. These EVs were reported to be different in their protein composition [10,14].

In the 1990s, Raposo et al. demonstrated that exosomes have effects on other cells by analyzing immune cells [15]. Exosomes derived from B lymphocytes induced antigen-specific MHC class II-restricted T cell responses, and this finding indicated that exosomes are involved in immune function. In 2006, it was also shown that mRNA is encapsulated in microvesicles derived from embryonic stem cells and delivered to hematopoietic progenitor cells [16]. Furthermore, in 2007, Valadi et al. reported that exosomes from the human mast cell line HMC-1 and the mouse mast cell line MC/9 contain approximately 1300 mRNAs and 121 microRNAs (miRNAs) that contribute to the exchange of genetic information between cells [17]. miRNAs are the most extensively studied class of short non-coding RNAs (ncRNAs) in the contents of EVs. In 2010, several important papers reporting on the function of EV-miRNAs were published. They showed that the transferred EV-miRNAs can be active in the recipient cell, modifying its phenotype [18,19,20]. Since that time, a number of studies have confirmed these preliminary observations. Currently, it has become clear that EVs are involved in a variety of biological phenomena and play critical roles in intracellular communication through the horizontal transfer of cellular cargoes such as proteins, DNA fragments, RNAs including mRNA and ncRNAs (miRNA, piRNA, and long ncRNA) and lipids [21]. This exchange of information between cells has been observed in a homeostatic state and in a variety of diseases. In particular, a large number of studies have reported associations with cancer in recent years.

The most common cause of death associated with cancer is metastasis. This process involves the growth and invasion of cancer cells, which leads to metastasis. The malignant transformation of cancer has been studied for a long time, and in 1889, Paget proposed the “seed and soil theory” [22]. It has been proposed that not only cancer cells but also the surrounding environment are important for metastasis. Since then, many studies have focused on the cancer microenvironment created by cancer cells and their surrounding cells. In addition to cancer cells, surrounding cells such as immune cells, inflammatory cells, vascular endothelial cells, and fibroblasts interact with each other through various molecules to form a cancer microenvironment in tumor tissue. Cytokines and adhesion molecules are representative examples of these molecules. In addition to these molecules, EVs, which contain pathogenic components, function as tools for cell-cell communication in tumor tissues and metastatic organs and are involved in the formation of the microenvironment at the primary tumor site and the metastatic site [23].

Some organs are common metastatic sites for cancer. Bone is the third most preferred site of metastasis, next to the lung and liver. Approximately 70% of patients with prostate cancer and breast cancer and 30–40% of those with lung cancer eventually develop metastasis in bone. Bone metastasis is generally incurable and dramatically affects patient quality of life. Bone metastasis can cause devastating complications called skeletal-related events (SREs), including hypercalcemia, pathological fractures, spinal compression, and bone pain, which result in a poor prognosis. Moreover, the median survival of bone metastasis from the time of diagnosis is low, including 12–52% for prostate cancer patients, 19–25% for breast cancer patients, and approximately 6–9% for lung cancer patients [24]. Therefore, there is an urgent need to investigate the mechanism of bone metastasis and provide strategies for the dramatic treatment of bone metastasis.

Although the mechanisms underlying bone metastasis are yet to be fully elucidated, increasing evidence suggests that EVs in the bone microenvironment significantly contribute to cancer bone tropism. In this review, we will discuss the remarkable effects of EVs, especially on the tumor microenvironment in bone.

## 2. Cancer Metastasis and EVs

Cancer-related EVs contribute to the formation and modulation of the microenvironment in the primary tumor site and the metastatic site. In addition to EVs from cancer cells, EVs from noncancerous cells, such as stromal cells, immune cells, inflammatory cells, vascular endothelial cells, and fibroblasts, have also been found to have significant impacts on cancer progression and remission. Many high-quality reports in recent years have revealed the interplay between cancer and the surrounding noncancerous cells and cells of distant metastatic sites through various forms of communication.

Cancer cells deliver EVs to surrounding noncancerous cells, altering the properties of surrounding cells to maintain a tumor microenvironment that is favorable for tumor cell survival. Kosaka et al. showed that EVs derived from breast cancer cell lines that have high metastatic abilities contain a large amount of miR-210, which induces angiogenesis, and they affect vascular endothelial cells. EV-miR-210 acts on vascular endothelial cells and inhibits EphrinA3 expression, inducing tumor angiogenesis. Furthermore, the action of these EVs also promotes lung metastasis [25]. Conversely, surrounding noncancerous cells also affect cancer cells through EV-mediated communication. Luga et al. reported that fibroblast-derived EVs increased breast cancer cell activity and promoted metastasis by activating the Wnt-PCP signaling system [26].

Moreover, cancer cells communicate with each other via EVs to gain metastatic capacity. Le et al. demonstrated that EVs from breast cancer cell lines with high metastatic potential could enhance the metastatic potential of breast cancer cell lines with low metastatic potential by providing miR-200 family members [27].

EVs secreted by cancer cells affect not only surrounding cells and fellow cancer cells but also distant cells. It has become clear that cancer cells use EVs to create an environment that facilitates tumor cell survival in the metastatic site before the cells metastasize. The environment in which this occurs is called the premetastatic niche. In a pioneering report, the authors found that EVs secreted by melanoma cells acted on cells in the bone marrow to guide them to the metastatic lung, causing the displaced cells in the bone marrow to form a premetastatic niche [28]. Since then, there have been several reports of the involvement of EVs in premetastatic niche formation. Recent investigations have revealed that EVs are part of the mechanism by which cells and organs are selected during the formation of such premetastatic niches, which is known as metastatic organ tropism. It has been suggested that differences in the expression patterns of integrins in EVs alter the cells that take up EVs, resulting in the formation of organ-specific premetastatic niches [29]. EVs derived from cancer cells profoundly influence the establishment of premetastatic niches, induce vascular leakage at premetastatic sites to help cancer cells colonize at metastatic sites and are also involved in metastatic organ selection.

## 3. Bone Microenvironment/Bone Remodeling and EVs

Before proceeding to the main topic of this review, bone metastasis and EVs, we will describe the normal bone microenvironment and EVs. The bone microenvironment consists of three main cells: osteoclasts, osteoblasts, and osteocytes. Moreover, bone marrow stromal cells (BMSCs), such as mesenchymal stem cells (MSCs), are also deeply associated with the formation of the bone microenvironment. These cells maintain the normal structure and homeostasis of the bone, exchanging various messengers with each other. Osteoclasts are derived from monocytes, differentiate, fuse during maturation and resorb existing bone. Osteoblasts are derived from MSCs in the bone marrow and are responsible for the formation of new bone.

Osteoclast differentiation occurs under the influence of macrophage colony-stimulating factor (M-CSF) and receptor activator of nuclear factor kappa-B ligand (RANKL), which are secreted by stromal cells, osteoblasts, osteocytes, and immune cells [30]. The binding of M-CSF to the cell-surface receptor colony-stimulating factor-1 receptor (c-fms) on osteoclast precursors promotes the proliferation and expression of the RANK receptor. The interaction between RANK and RANKL leads to osteoclast differentiation and sustains the survival and activity of osteoclasts. Transforming growth factor beta (TGF-β), fibroblast growth factor (FGF), insulin growth factor (IGF), platelet-derived growth factor (PDGF), and bone morphogenetic proteins (BMPs) are released from the bone matrix during osteoclast bone resorption. These factors stimulate the differentiation of MSCs into osteoblasts [30]. It is also known that osteoclasts can autonomously induce osteoblast differentiation and activation by secreting factors such as sphingosine-1-phosphate (S1P) [31,32]. S1P is a metabolic product of sphingolipids, a component of biological membranes, and promotes the migration of target cells. Other factors secreted by osteoclasts have been reported as factors that regulate osteoblasts. Bone resorbing, mature osteoclasts secrete collagen triple helix repeat containing 1 (Cthrc1). Cthrc1, which is a protein with a collagen-like sequence that is induced when blood vessels are damaged, has been shown to promote late differentiation of osteoblasts [33]. Tartrate-resistant acid phosphatase (TRAcP) can also stimulate osteoblasts, inducing an increase in alkaline phosphatase (ALP) activity in vivo [34]. In turn, osteoblasts regulate osteoclastogenesis by the release of M-CSF, RANKL, and the RANKL receptor decoy osteoprotegerin (OPG) [30]. Under normal adult physiological conditions, these osteoclastic and osteoblastic activities are exquisitely balanced, which is called bone remodeling [35]. Moreover, during bone remodeling, the conjugation mechanism of prior bone resorption by osteoclasts and subsequent bone formation by osteoblasts is called coupling.

Other models of coupling mechanisms have been proposed. Zhao et al. focused on the increasing expression of EphrinB2 during osteoclast differentiation and found that osteoclast EphrinB2 affects the receptor EphB4 on osteoblasts to promote osteoblast differentiation and, at the same time, suppress osteoclast differentiation in vitro and in vivo [36]. It has also been suggested that a different subfamily, EphrinA2/EphA2, inhibits osteogenesis and promotes osteoclast differentiation early in the bone remodeling process [37]. Ephrin and its receptor Eph are known as membrane-type ligands and receptors and play important roles in nerve cell migration and axonal guidance. Semaphorin, which is important for neuroaxonal guidance similar to that of ephrin, is also known to be an important factor in the crosstalk between osteoclasts and osteoblasts. Semaphorin 4D is upregulated upon osteoclast differentiation and suppresses osteogenesis [38]. Until recently, these cytokines and cell adhesion molecules were thought to be central players that regulate the bone microenvironment.

**Figure 1:**
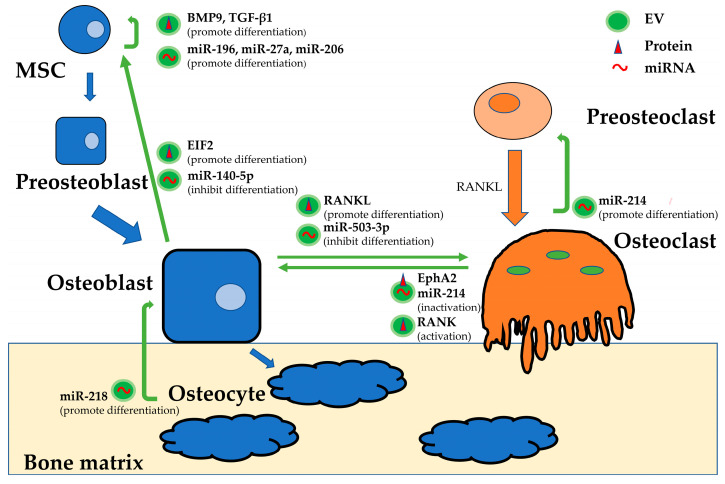
The main functions of EV cargoes involved in bone remodeling. Opposing EV functions depend on their various contents, even though EVs are produced by the same type of cell. The regulation of bone remodeling by exchanging EV contents is extremely complex.

Recently, however, EVs have been shown to play a very important role in maintaining this beneficial cycle as messengers of cell-to-cell exchange, in addition to the cytokines and cell adhesion molecules [39]. If the process of bone metastasis in tumors is a process of switching from a beneficial cycle to a vicious cycle, understanding the EVs involved in this beneficial cycle could enable elucidation of the mechanism of bone metastasis. Here, we describe the characteristics and roles of EVs derived from each of the cells in normal bone remodeling (Figure 1).

Protein characterization of EVs isolated from osteoclasts revealed that they express tumor susceptibility gene (TSG) 101, heat shock protein (HSP) 70, β-actin, CD 63, and epithelial cell adhesion molecule (EpCAM) [40]. Sun et al. demonstrated that miR-214-enriched osteoclast-derived EVs could be transferred into osteoblasts to inhibit their activity via EphrinA2/EphA2 [41]. A previous study by Wang et al. identified that miR-214 inhibited osteoblast function by targeting ATF4 [42], while further experiments demonstrated that miR-214 promoted osteoclastogenesis through the PI3K-Akt pathway by targeting PTEN [43]. Therefore, miR-214-containing EVs from osteoclasts may have multiple roles that promote bone destruction. Ikebuchi et al. showed that RANK in membrane vesicles secreted by maturing osteoclasts binds to RANKL on the surface of osteoblasts and activates the RANKL reverse signal, ultimately activating the transcription factor Runx2, which promotes bone formation [44].

Proteomics analysis of osteoblast-derived EVs revealed that proteins from osteoblast EVs are closely involved in the eukaryotic initiation factor (EIF) 2 pathway [45]. EIF2 is involved in BMP2-induced osteoblast differentiation, playing a central role in the development of the skeletal system [46,47]. EIF2 in osteoblast-derived EVs induces MSCs to differentiate into osteoblasts [45]. That is, osteoblasts establish a positive feedback mechanism that promotes bone growth through EVs released by the osteoblast itself. EVs isolated from the mineralizing MC3T3-E1 mature osteoblast cell line promoted osteoblastic differentiation of osteoblast precursor ST2 cells, changing the miRNA profile and activating pathways that play key roles in the differentiation (the Wnt, insulin, and TGF-β signaling pathways) and function (the calcium signaling pathway) of osteoblasts [48]. Conversely, Hwang et al. reported that miR-140-5p, which is enriched in osteoblast-derived EVs, suppressed osteoblastic differentiation of MSCs by inhibiting BMP2 expression [49]. Solberg et al. showed that osteoblast—and osteocyte-derived lysosomal membrane protein (LAMP) 1-positive EVs also contained TRAcP, RANKL and OPG, which are critical for osteoclast differentiation [50]. As Deng et al. identified, RANKL-rich EVs derived from osteoblasts stimulated osteoclast formation, inducing the nuclear translocation of nuclear factor of activated T cells (NFATc1) [51]. On the other hand, mineralized osteoblasts can secrete EVs containing miR-503-3p, which is able to inhibit osteoclast differentiation by reducing RANK expression [52]. Sato et al. reported that osteocyte-derived EVs containing miR-218 could induce the downregulation of sclerostin, thus promoting osteoblast differentiation [53].

EVs from BMSCs can also participate in bone remodeling by directly regulating osteoblast proliferation and activity [54]. MSC-derived EVs express the characteristic markers CD13, CD29, CD44, CD73 and CD105 [55] and have been shown to upregulate the expression of the growth factors BMP9 and TGF-β1 [56], both of which induce the osteogenic differentiation of MSCs [57]. MSC-derived EVs bind and tether extracellular matrix (ECM) proteins, such as type I collagen and fibronectin, to the bone surface and biomaterials. This function enables MSC-derived EVs to induce the differentiation of MSCs into osteogenic lineages [58]. Vallabhaneni et al. also characterized the contents of EVs from MSCs and found tumor-supportive miRNAs, including miR-21 and miR-34a, proteins such as PDGFR-β, tissue inhibitor of matrix metalloproteinase (TIMP) 1, and TIMP2, bioactive lipids and metabolites [59]. Regarding miRNAs, miR-196, miR-27a, and miR-206 are particularly enriched in EVs derived from MSCs and can stimulate osteogenic differentiation in vitro, and the treatment of calvarial bone defects with MSC-derived EVs promoted bone regeneration in vivo [54]. As seen above, it has been reported that the many different EV functions depend on their various contents, even though EVs are produced by the same cells. It is thought that the regulation of bone remodeling by exchanging the contents of EVs would be extremely complex. However, there may be a critical factor associated with bone metastasis hidden in these various factors, as previously mentioned. We should continue to make efforts to understand the entire mechanism of bone remodeling.

## 4. Bone Metastasis and EVs

There have been a considerable number of reports on EVs in the development and dissemination of cancer. However, the role of EVs in the development and exacerbation of bone metastasis has not been widely reported. Bone metastasis is said to be a complex cascade of processes [60]. The harmonious beneficial cycle of cells forming the bone microenvironment can easily become fertile soil for secondary tumors, and cancer cells can turn this beneficial cycle into a vicious one. First, tumor cells can travel into the bone through specific migration and invasion processes. Second, these tumor cells gain bone-like characteristics and reach the bone marrow. Finally, tumor cells interact with the cells that make up the bone structure to hijack physiological bone metabolic pathways and create a microenvironment that is suitable for invasion and proliferation in the bone.

**Figure 2:**
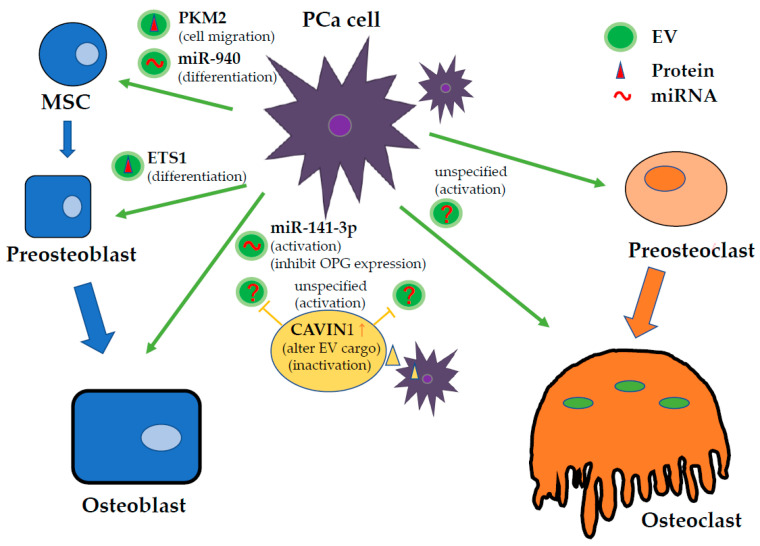
The main functions of EV cargo derived from PCa cells in the bone tumor microenvironment. EVs secreted by PCa change the beneficial bone remodeling cycle into a vicious cycle, affecting bone cells. Recent investigations reported that EVs derived from bone cells also affect cancer cell viability. It is thought that EV functions in the bone tumor microenvironment are very complex.

Unfortunately, the specific EV components that determine bone metastasis have not been identified, but EVs are involved in any of these processes, and EVs associated with cancer cells are key players in carrying cargo that adversely affects bone metabolism and obstructs the vicious cycle in the bone microenvironment. The goal of this review is to consolidate the current findings on bone metastasis-related EVs, which are listed in a table. In the following sections, we summarize the research findings with a focus mainly on prostate cancer (Figure 2), breast cancer, and lung cancer, which are the major cancers associated with bone metastasis (Table 1).

### 4.1. Prostate Cancer

Bone is the most frequent metastatic target in men with advanced prostate cancer (PCa). However, the mechanisms that favor to the more frequent development of bone metastasis than soft tissue metastasis in PCa are not well defined. Dai et al. reported that primary PCa cells educate the bone marrow to establish a premetastatic niche through primary PCa cell-derived EV-mediated transfer of pyruvate kinase M2 (PKM2) into BMSCs and the subsequent upregulation of CXCL12, a chemokine involved in cell migration [64]. This report suggests a mechanism through which the primary tumor crosstalks with the bone microenvironment to establish a premetastatic niche.

It is also well known that PCa mainly exhibits osteosclerotic bone metastasis. In fact, bone metastasis in PCa has both osteogenic and osteolytic aspects. EVs involved in PCa may therefore affect osteoclasts and osteoblasts. At the leading edge of tumor invasion in bone metastasis, bone resorption occurs first and is thought to be necessary for subsequent bone formation. First, bone resorption is facilitated by mature osteoclasts. Inder et al. showed that EVs derived from the human PCa cell line PC3 were internalized by murine osteoclast precursor RAW264.7 cells and primary human osteoblasts (hOBs) in vitro, stimulating osteoclastogenesis 37-fold and hOB proliferation 1.5-fold, respectively. This phenomenon was not observed in PC3 cells transfected with a vector containing caveolae associated protein1 (CAVIN1), also known as polymerase I and transcript release factor (PTRF). The authors previously demonstrated that CAVIN1 expression suppressed PC3 tumor growth and metastasis in vivo concomitant with changes in the PC3-EV proteome. They showed that CAVIN1 was not detected in EVs, and then Cavin1-mediated pathways could attenuate metastatic PCa through its associated EV cargo recruitment [61]. On the other hand, Karlsson et al. showed that EVs isolated from the murine PCa cell line TRAMP-C1 inhibited the development of the osteoclast lineage, decreasing the expression of established markers for osteoclast fusion and differentiation, including dendritic cell-specific transmembrane protein (DC-STAMP), TRAP, cathepsin K (CTSK), and matrix metallopeptidase (MMP)-9 [62]. The exact opposite results of these two reports may be due to differences in the PCa cell lines; PC3 cells predominantly cause osteogenic metastasis, and TRAMP-C1 cells predominantly cause osteolytic metastasis. It is significant to note that the nature of the cell lines could decisively influence the results in PCa research.

Subsequent bone formation is performed by osteoblasts. Itoh et al. reported that EVs derived from either the metastatic PCa cell line PC3 or DU145 cell cultures significantly promoted osteoblast differentiation, while EVs from the androgen-sensitive human prostate adenocarcinoma cell line LNCaP did not have this effect. Hormone refractory PCa cell-derived EVs containing ETS1, which is an osteoblast differentiation-associated transcription factor, were transferred into osteoblasts and induced differentiation [63]. Ye et al. demonstrated that EVs derived from the metastatic PCa cell line MDA PCa 2b, which contain miR-141-3p, promoted osteoblasts, leading to increased expression of the osteoclast inhibitory factor OPG [65]. miR-141-3p suppressed the protein levels of the target gene DLC1, indicating its functional significance in activating the p38 MAPK pathway. p38 MAPK plays an important role in osteoblast activity, which can significantly increase alkaline phosphatase activity and calcium deposition. Mice injected with EVs containing miR-141-3p-mimic developed apparent osteoblastic bone metastasis. Hashimoto et al. showed that hsa-miR-940, which was highly enriched in EVs released by PCa cells, promoted osteogenic differentiation of human MSCs in vitro, targeting ARHGAP1 and FAM134A and inducing extensive osteoblastic lesions in the bone metastatic microenvironment in vivo. Their study indicated that the osteoblastic or osteolytic phenotype of bone metastasis can be induced by miRNAs secreted by cancer cells in the bone microenvironment [66]. Probert et al. found that treatment with PC3-derived EVs increased osteoblast viability. By using techniques to track RNA, the authors also identified the delivery of a set of PCa-RNAs to osteoblast via EVs, which affected their endogenous transcript abundance. This group showed the contribution of the RNA component of EV cargo, providing evidence to support EV communication via RNA molecules as a potential strategy for mediating bone metastasis [67].

Furthermore, EVs derived from osteoblasts also have an effect on prostate cancer cells. Morhayim et al. demonstrated that osteoblasts promote PCa cell (PC3) proliferation by the level of mineralization stage-specific proteins in osteoblast-secreted EVs. This group claimed that the stages of differentiation and the mineralization condition were also among the factors that influenced the nature and abundance of EV cargo. Bioinformatics analyses of osteoblast-derived EV proteomes and EV-regulated PCa gene expression profiles showed that these factors converged on pathways involved in cell survival and growth. This finding was verified by in vitro proliferation assays in which osteoblast uptake of EVs led to a 2-fold increase in PC3 cell growth compared to that of the cell-free culture medium-derived vesicle controls [68].

### 4.2. Breast Cancer

It is well known that breast cancer (BCa) can also easily translocate into the bone and mainly exhibits osteolytic metastasis. Unexpectedly, however, there have been few reports on the exchange of EVs between BCa cells and bone cells, whereas there have been many reports on the involvement of EVs in brain or lung metastasis.

Tiedemann et al. showed that BCa cell-derived EVs containing L-plastin mediates osteoclast activation, which in turn created an osteolytic microenvironment that was favorable to breast cancer growth [69]. Liu et al. reported that EVs derived from the metastatic breast cancer cell line MDA-MB-231 transferred miR-218 to regulate osteoblast activity, which suppressed osteoblast type I collagen production and osteoblast differentiation. Hashimoto et al. found that inducing miR-940 overexpression in the BCa cell line MDA-MB-231 induced extensive osteoblastic lesions in mouse models in vivo by facilitating the osteogenic differentiation of host mesenchymal cells. The authors demonstrated that the transfer of EVs from cancer to stromal cells was responsible for osteoblastic lesion induction [66].

There have been some other reports examining EVs derived from BCa cells, which were not limited to those specific to bone metastasis. Wen et al. demonstrated that EVs from highly metastatic BCa cells potentially contributed to establishing a premetastatic niche that was also able to promote metastasis by altering immune cell activity. BCa-derived EVs can suppress CD8 and CD4 T-cell proliferation and reduce NK cytotoxic activity against target tumor cells. This phenomenon also occurs in the bone tumor microenvironment. The acquisition of the ability of tumor cells to escape immune surveillance is also an important factor that promotes metastasis [70].

Most of the reports on the involvement of EVs secreted by BCa cells in bone focus on the dormancy of BCa cells in the bone marrow. Cancer dormancy is generally defined as the arrest of tumor growth in the primary or metastatic site due to cancer cell quiescence. During this period, cancer cells acquire drug resistance until stimulated to awaken, ultimately causing cancer relapse and metastasis. Ono et al. described that coculturing bone metastatic MDA-MB-231 cells with bone marrow-mesenchymal stem cells (BM-MSCs) suppressed tumor cell proliferation and sensitivity to chemotherapy, suggesting the acquisition of a dormant phenotype by tumor cells [71]. Interestingly, the same result was observed by culturing tumor cells with BM-MSC-derived EVs due to their cargo containing miR-23b. Furthermore, Bliss et al. demonstrated that breast cancer cells stimulate the release of miR-222/223-containing EVs from MSCs, which then promote quiescence in a subset of cancer cells and affect drug resistance [72].

### 4.3. Lung Cancer

Lung cancer (LungCa) also has a tendency to induce osteolytic metastasis. Non-small cell lung cancer (NSCLC) cells are said to release factors that alter bone remodeling and increase osteoclast activity through shifting the normal balance of RANKL, RANK and OPG [73].

Taverna et al. showed that NSCLC cells secrete EVs containing epidermal growth factor receptor (EGFR) ligand and amphiregulin (AREG), which stimulate osteoclastogenesis [74]. The relevance of these data was supported by the fact that patient-derived EVs were able to modulate osteoclastogenesis in human osteoclast precursors. AREG knockdown, neutralization with AREG antibodies, and cotreatment with NSCLC-EVs and the epidermal growth factor receptor–tyrosine kinase inhibitor erlotinib reversed EV-induced osteoclast differentiation. Moreover, Xu et al. found that treatment of bone marrow-derived monocytes with adenocarcinoma-derived EVs promoted osteoclast formation by shuttling miR-21, which in turn inhibited Pdcd4, a transcription factor involved in osteoclastogenesis [75].

In contrast, Valencia et al. showed that EVs isolated from the miR-192-overexpressing metastatic human adenocarcinoma A549 cell line could reduce in vivo osteolytic lesions. Altering the cargo of cancer cell-derived EVs via the overexpression of a single anti-angiogenic miRNA (miR-192) repressed tumor-induced angiogenesis, which led to a reduction in the number of bone metastatic lesions [76].

Cancer bone metastasis has been studied in various types of cancers. EV research in primary bone tumors, such as multiple myeloma and osteosarcoma, is also active. These studies could provide crucial hints in the field of bone metastasis. It is important to discover the characteristics that are common to all types of cancer and are specific to bone metastasis. This will lead to future critical treatments that provide a glimmer of hope for the many patients suffering from bone metastasis.

## 5. Therapeutic Perspectives of EVs

Unfortunately, to the best of our knowledge, there are currently no reports of the use of EVs to treat bone metastasis in cancer. However, as the relationship between EVs and bone metastasis becomes clearer, it is possible that more research will be conducted on therapies specific to bone metastasis. In this section, the basic idea of the therapeutic application of EVs and the latest trends will be discussed. There are two main ways in which EVs can be used to reduce the progression of cancer, including bone metastasis: using EVs as DDS carriers or targeting EVs themselves as therapeutic targets [77]. In the following sections, we will discuss the EV-related treatments, which are broadly divided into these two categories.

### 5.1. The Use of EVs as DDS Carriers

In recent years, EVs have been actively investigated as DDS agents [78]. EVs are naturally present in body fluids and are stable in a variety of environments. In addition, EVs are selective carriers for specific target cells due to their unique membrane proteins and lipids that can bind to specific receptors, making them highly efficient for delivery [79]. Furthermore, EVs are a size that is difficult to filter and expel in the kidney, and in tumor and inflammatory tissues, they have a tendency to flow out of the blood vessels and passively accumulate in the tissues due to increased vascular permeability (enhanced permeation and retention effect; EPR effect) [80]. These properties make EVs the most suitable vehicles for transporting drugs and nucleic acids.

Among EVs, especially small EVs, their small size and long half-life in circulation make them ideal drug-delivery vehicles [81]. Thus, Tian et al. isolated sEVs from the immature dendritic cells (imDCs) of mice and loaded them with the chemotherapeutic drug doxorubicin (DXR) by electroporation [82]. The imDCs were also designed to express the EV membrane protein Lamp2b fused to an αv integrin-specific iRGD peptide (CRGDKGPDC) to increase interactions with αv-expressing target cells such as MDA-MB-231 cells. iRGD DXR-sEVs were effective in suppressing MDA-MB-231 cells in an orthotopic mouse model of breast cancer, showing high efficiency and low toxicity compared to those of free DXR treatment.

Ohno et al. demonstrated that EVs can efficiently deliver miRNAs to breast cancer cells expressing epidermal growth factor receptor (EGFR) [83]. The GE11 peptide, which binds specifically to EGFR and is less mitogenic than EGF, was overexpressed on the surface of EVs. These modified EVs were able to deliver let-7a in a manner that was dependent on EGFR expression. These results suggest that EVs can be used therapeutically to target EGFR-expressing cancer tissues and deliver nucleic acid drugs.

In addition, in recent years, bovine milk-derived EVs have become a hot topic as drug delivery vehicles [84,85]. Bovine-milk-derived EVs are inexpensive and easy to extract in large quantities and have low toxicity. However, the low encapsulation rate is the most significant remaining issue. To use EVs as DDSs, it is important to encapsulate the drugs within the EVs, store the EVs, and establish organ tropism. As far as we know, no appropriate method for storing EVs as DDSs and no critical factors for the specific accumulation of EVs in the bone have been reported. Further development of bone-related EV research is needed to use EVs as DDSs to bone.

### 5.2. EV-Targeted Therapy

There are three possible therapeutic strategies for targeting EVs themselves: inhibition of EV production, elimination of circulating EVs, and inhibition of EV absorption [77].

Some reports have described the efficacy of inhibiting EV production to suppress cancer progression. One of the notable targets in this strategy is ceramide, a type of lipid identified in the synthetic pathway that is synthesized by neutral sphingomyelinase (nSMase) 2. Previous attempts have been made to inhibit ceramide production by knocking down nSMase2 or using its inhibitor GW4869 [18,86,87]. Kosaka et al. demonstrated that knockdown of nSMase2 reduced EV secretion and miR-210 transcription, leading to the inhibition of angiogenesis and metastasis in a xenograft mouse model [25]. Other molecules, such as RAB27A/B, TSG101, and TSAP6, have also been shown to be involved in the secretion of EVs from cancer cells.

Eliminating circulating EVs could be a relatively new treatment strategy in cancer patients. A method to remove EVs using cancer-specific antigens has been reported [88]. In this study, the cancer-specific EV surface protein HER2 was successfully used to remove HER2-positive EVs derived from circulating cancer cells. HER2-expressing EVs have been shown to diminish therapeutic effects and are associated with cancer progression [89], and selective removal of HER2-expressing EVs could be a novel strategy for BCa treatment. Nishida et al. found that cancer-derived EVs were disrupted by macrophages in response to antibody therapy with anti-CD9 or anti-CD63 in a metastatic BCa cancer mouse model and drastically suppressed metastatic cells [90].

It has been recently reported that the absorption of EVs is induced by various mechanisms, such as clathrin-mediated endocytosis, phagocytosis, micropinocytosis, and fusion of the plasma and endosomal membranes [91], and involves various molecules. As EVs can be directed to specific target cells, targeting the molecules involved in their reception allows for treatments with high specificity and low side effects.

Although therapeutic strategies targeting EVs released from cancer cells are effective, several problems remain before clinical application is feasible. Molecules identified in previous studies play important roles in multiple cellular biological phenomena and can adversely affect normal cell functions when downregulated in normal cells. In other words, the identification of cancer-specific molecules is essential for the future development of EV-targeted anticancer drugs. As we have seen in this review, various factors have complicated mechanisms in the bone microenvironment. The search for specific factors that determine bone metastasis is underway. Some of these factors may have be potential targets for EV therapy.

## 6. Conclusions

It is well known that the fossil record, which includes skeletal samples of human beings, is used in anthropology. Skeletons are special structures with universality and stationarity that transcend time and space. However, bones are also fluid structures that are constantly metabolized. It is undoubtedly true that bone metastasis is unique and distinct from visceral metastasis. Researchers worldwide have been struggling to understand the mechanisms of bone metabolism and overcome or prevent bone metastasis.

Currently, the most prevalent treatment for bone metastasis of PCa, BCa, and LungCa involves the bone modifying agents bisphosphonates and denosumab. Bisphosphonates are antiresorptive agents that block pathological bone resorption by inhibiting osteoclast activation and function [92]. Denosumab, a monoclonal antibody against RANKL, also received regulatory approval for preventing SREs in patients with bone metastasis from solid tumors. Although the mechanisms of action of bisphosphonates and denosumab are different, both agents interrupt the vicious cycle of increased osteolysis coupled with increased tumor growth. Furthermore, it has been suggested that bone-modifying agents may delay the progression of bone lesions and help delay the development of skeletal and other metastases [92,93,94], potentially by making the bone microenvironment less conducive to tumor growth. These drugs have been developed as a result of continuous bone research, and combined treatment with these bone-modifying agents and radiation has become a common strategy for treating bone metastasis. Unfortunately, however, this treatment strategy is only partially effective and is palliative treatment, not curative. The development of more powerful and safe treatments is needed in the clinic. The process of bone metastasis of cancer is very complex. Future therapeutic strategies for treating bone metastasis will not be a single method but combinations of several drugs or modalities that will block multiple targets and pathways as a part of the communication network in the bone-tumor microenvironment.

Similar to that of cytokines and cell adhesion molecules, the function of EVs in the cell-to-cell communication network is becoming clearer. There is no longer any doubt that EVs are key players in that network. Emerging evidence on EV functions in bone metastasis may allow the discovery of novel treatments. EV research is still in the developmental stage. Many problems remain before clinical application can be achieved; however, we can see the potential of EVs as powerful and promising tools for cancer therapy. It is quite possible that the next emerging therapy for bone metastasis would be a drug targeting EVs or a drug using EVs. In the near future, EV-based therapy will help to dramatically overcome bone metastasis. Above all, we need to understand the messages exchanged between the cells that form the tumor microenvironment in bone. We hope this review will help with that first step.

## Figures and Tables

**Table 1:** List of molecular factors, cell targets and functions involved in EVs in bone metastatic microenvironment.

Tumor	Origin of EVs	Molecular Factors	Target	Function	Ref.
PCa	PCa cells (PC3)	unspecified	osteoclasts	promotion of osteoclast differentiation	[50]
	PCa cells (PC3)	unspecified	osteoblasts	promotion of osteoblasts proliferation	[50]
	PCa cells (PC3)	CAVIN1 (not contained in EVs)	osteoclasts	inhibition of osteoclast differentiation by altering EV cargo	[50]
	PCa cells (PC3)	CAVIN1 (not contained in EVs)	osteoblasts	inhibition of osteoblast proliferation by altering EV cargo	[50]
	PCa cells (TRAMP-C1)	unspecified	osteoclasts	inhibition of osteoclast fusion and differentiation	[51]
	PCa cells (PC3, DU145)	ETS1	osteoblasts	promotion of osteoblast differentiation	[52]
	PCa cells (MDA-PCa-2b)	miR-141-3p	osteoblasts	promotion of osteoblasts activity and increase OPG (osteoclast inhibitory factor) expression	[53]
	PCa cells (C4, C4-2, C4-2B)	miR-940	osteoblasts	promotion of osteoblast differentiation targeting ARHGAP1 and FAM134A	[54]
	PCa cells (PC3)	miRNA (i.e., miR-21) and miRNA (i.e., CSF-1)	osteoblasts	promotion of osteoblast viability	[55]
	osteoblasts	mineralization stage-specific protein	PCa cells (PC3)	promotion of PCa cell proliferation	[56]
BCa	BCa cells (MDA-MB-231)	L-plastin	osteoclasts	promotion of osteoclast activation	[57]
	BCa cells (MDA-MB-231)	miR-218	osteoblasts	inhibition of osteoblast defferentiation	[54]
	BCa cells (EO771)	unspecified	CD8 CD4 T-cells, NK cells	inhibitoin of immune cell activity against target tumor cells	[58]
	BM-MSCs	miR-23b	BCa cells (MDA-MB-231)	suprression of BCa cell proliferation, as well as sensitivity to chemotherapy and acquisition of a dormant phenotype	[59]
	MSCs	miR-222/223	BCa cells (MDA-MB-231)	suprression of BCa cell proliferation as well as sensitivity to chemotherapy	[60]
LungCa	NSCLC cells (CRL-2868, A549)	AREG	osteoclasts	promotion of osteoclast differentiation	[61]
	NSCLC cells (A549)	miR-21	osteoclasts	promotion of osteoclast differentiation	[62]
	NSCLC cells (A549)	miR-192	endthelial cells	promotion of osteoclast differentiation	[63]

## References

[1] Chargaff E., West R. (1946). The biological significance of the thromboplastic protein of blood. J. Biol. Chem..

[2] Wolf P. (1967). The Nature and Significance of Platelet Products in Human Plasma. Br. J. Haematol..

[3] Anderson H.C. (1969). Vesicles associated with calcification in the matrix of epiphyseal cartilage. J. Cell Biol..

[4] Crawford N. (1971). The Presence of Contractile Proteins in Platelet Microparticles Isolated from Human and Animal Platelet-free Plasma. Br. J. Haematol..

[5] Dalton A.J. (1975). Microvesicles and Vesicles of Multivesicular Bodies Versus “Virus-Like” Particles. J. Natl. Cancer Inst..

[6] Dvorak H.F., Quay S.C., Orenstein N.S., Dvorak A.M., Hahn P., Bitzer A.M., Carvalho A.C. (1981). Tumor shedding and coagulation. Science.

[7] Stegmayr B., Ronquist G. (1982). Promotive effect on human sperm progressive motility by prostasomes. Urol. Res..

[8] Pan B.T., Johnstone R.M. (1983). Fate of the transferrin receptor during maturation of sheep reticulocytes in vitro: Selective externalization of the receptor. Cell.

[9] Johnstone R.M., Adam M., Hammond J.R., Orr L., Turbide C. (1987). Vesicle formation during reticulocyte maturation. Association of plasma membrane activities with released vesicles (exosomes). J. Biol. Chem..

[10] Théry C., Witwer K.W., Aikawa E., Alcaraz M.J., Anderson J.D., Andriantsitohaina R., Antoniou A., Arab T., Archer F., Atkin-Smith G.K. (2018). Minimal information for studies of extracellular vesicles 2018 (MISEV2018): A position statement of the International Society for Extracellular Vesicles and update of the MISEV2014 guidelines. J. Extracell. Vesicles.

[11] Witwer K.W., Théry C. (2019). Extracellular vesicles or exosomes? On primacy, precision, and popularity influencing a choice of nomenclature. J. Extracell. Vesicles.

[12] György B., Szabó T.G., Pásztói M., Pál Z., Misják P., Aradi B., László V., Pállinger É., Pap E., Kittel Á. (2011). Membrane vesicles, current state-of-the-art: Emerging role of extracellular vesicles. Cell. Mol. Life Sci..

[13] Raposo G., Stoorvogel W. (2013). Extracellular vesicles: Exosomes, microvesicles, and friends. J. Cell Biol..

[14] Kowal J., Arras G., Colombo M., Jouve M., Morath J.P., Primdal-Bengtson B., Dingli F., Loew D., Tkach M., Théry C. (2016). Proteomic comparison defines novel markers to characterize heterogeneous populations of extracellular vesicle subtypes. Phys. Sci..

[15] Raposo G., Nijman H.W., Stoorvogel W., Leijendekker R., Harding C.V., Melief C.J.M., Geuze H.J. (1996). B lymphocytes secrete antigen-presenting vesicles. J. Exp. Med..

[16] Ratajczak J., Miekus K., Kucia M., Zhang J., Reca R., Dvorak P., Ratajczak M.Z. (2006). Embryonic stem cell-derived microvesicles reprogram hematopoietic progenitors: Evidence for horizontal transfer of mRNA and protein delivery. Leukemia.

[17] Valadi H., Ekström K., Bossios A., Sjöstrand M., Lee J.J., Lötvall J.O. (2007). Exosome-mediated transfer of mRNAs and microRNAs is a novel mechanism of genetic exchange between cells. Nat. Cell Biol..

[18] Kosaka N., Iguchi H., Yoshioka Y., Takeshita F., Matsuki Y., Ochiya T. (2010). Secretory mechanisms and intercellular transfer of microRNAs in living cells. J. Biol. Chem..

[19] Pegtel D.M., Cosmopoulos K., Thorley-Lawson D.A., Van Eijndhoven M.A.J., Hopmans E.S., Lindenberg J.L., De Gruijl T.D., Würdinger T., Middeldorp J.M. (2010). Functional delivery of viral miRNAs via exosomes. Proc. Natl. Acad. Sci. USA.

[20] Zhang Y., Liu D., Chen X., Li J., Li L., Bian Z., Sun F., Lu J., Yin Y., Cai X. (2010). Secreted Monocytic miR-150 Enhances Targeted Endothelial Cell Migration. Mol. Cell.

[21] Yáñez-Mó M., Siljander P.R.M., Andreu Z., Zavec A.B., Borràs F.E., Buzas E.I., Buzas K., Casal E., Cappello F., Carvalho J. (2015). Biological properties of extracellular vesicles and their physiological functions. J. Extracell. Vesicles.

[22] Paget S. (1889). The Distribution of Secondary Growths in Cancer of the Breast. Lancet.

[23] Naito Y., Yoshioka Y., Yamamoto Y., Ochiya T. (2017). How cancer cells dictate their microenvironment: Present roles of extracellular vesicles. Cell. Mol. Life Sci..

[24] Selvaggi G., Scagliotti G.V. (2005). Management of bone metastases in cancer: A review. Crit. Rev. Oncol. Hematol..

[25] Kosaka N., Iguchi H., Hagiwara K., Yoshioka Y., Takeshita F., Ochiya T. (2013). Neutral sphingomyelinase 2 (nSMase2)-dependent exosomal transfer of angiogenic micrornas regulate cancer cell metastasis. J. Biol. Chem..

[26] Luga V., Zhang L., Viloria-Petit A.M., Ogunjimi A.A., Inanlou M.R., Chiu E., Buchanan M., Hosein A.N., Basik M., Wrana J.L. (2012). Exosomes mediate stromal mobilization of autocrine Wnt-PCP signaling in breast cancer cell migration. Cell.

[27] Le M.T.N., Hamar P., Guo C., Basar E., Perdigão-Henriques R., Balaj L., Lieberman J. (2014). MiR-200-containing extracellular vesicles promote breast cancer cell metastasis. J. Clin. Investig..

[28] Peinado H., Alečković M., Lavotshkin S., Matei I., Costa-Silva B., Moreno-Bueno G., Hergueta-Redondo M., Williams C., García-Santos G., Ghajar C.M. (2012). Melanoma exosomes educate bone marrow progenitor cells toward a pro-metastatic phenotype through MET. Nat. Med..

[29] Hoshino A., Costa-Silva B., Shen T.L., Rodrigues G., Hashimoto A., Mark M.T., Molina H., Kohsaka S., Di Giannatale A., Ceder S. (2015). Tumour Exosome Integrins Determine Organotropic Metastasis. Nature.

[30] Fattore A. (2012). Del Bone cells and the mechanisms of bone remodelling. Front. Biosci..

[31] Boyce B.F., Xing L. (2006). Osteoclasts, no longer osteoblast slaves. Nat. Med..

[32] Ryu J., Kim H.J., Chang E.-J., Huang H., Banno Y., Kim H.-H. (2006). Sphingosine 1-phosphate as a regulator of osteoclast differentiation and osteoclast–osteoblast coupling. EMBO J..

[33] Takeshita S., Fumoto T., Matsuoka K., Park K.A., Aburatani H., Kato S., Ito M., Ikeda K. (2013). Osteoclast-secreted CTHRC1 in the coupling of bone resorption to formation. J. Clin. Investig..

[34] Del Fattore A., Fornari R., Van Wesenbeeck L., de Freitas F., Timmermans J.-P., Peruzzi B., Cappariello A., Rucci N., Spera G., Helfrich M.H. (2007). A New Heterozygous Mutation (R714C) of the Osteopetrosis Gene, Pleckstrin Homolog Domain Containing Family M (With Run Domain) Member 1 (PLEKHM1), Impairs Vesicular Acidification and Increases TRACP Secretion in Osteoclasts. J. Bone Miner. Res..

[35] Florencio-Silva R., Rodrigues Da G., Sasso S., Sasso-Cerri E., Simões M.J., Cerri P.S. (2015). Biology of Bone Tissue: Structure, Function, and Factors That Influence Bone Cells. BioMed. Res. Int..

[36] Zhao C., Irie N., Takada Y., Shimoda K., Miyamoto T., Nishiwaki T., Suda T., Matsuo K. (2006). Bidirectional ephrinB2-EphB4 signaling controls bone homeostasis. Cell Metab..

[37] Irie N., Takada Y., Watanabe Y., Matsuzaki Y., Naruse C., Asano M., Iwakura Y., Suda T., Matsuo K. (2009). Bidirectional signaling through EphrinA2-EphA2 enhances osteoclastogenesis and suppresses osteoblastogenesis. J. Biol. Chem..

[38] Negishi T.-K., Shinohara M., Komatsu N., Bito H., Kodama T., Friedel R.H., Takayanagi H. (2011). Suppression of bone formation by osteoclastic expression of semaphorin 4D. Nat. Med..

[39] Xie Y., Chen Y., Zhang L., Ge W., Tang P. (2017). The roles of bone-derived exosomes and exosomal microRNAs in regulating bone remodelling. J. Cell. Mol. Med..

[40] Li D., Liu J., Guo B., Liang C., Dang L., Lu C., He X., Cheung H.Y.S., Xu L., Lu C. (2016). Osteoclast-derived exosomal miR-214-3p inhibits osteoblastic bone formation. Nat. Commun..

[41] Sun W., Zhao C., Li Y., Wang L., Nie G., Peng J., Wang A., Zhang P., Tian W., Li Q. (2016). Osteoclast-derived microRNA-containing exosomes selectively inhibit osteoblast activity. Cell Discov..

[42] Wang X., Guo B., Li Q., Peng J., Yang Z., Wang A., Li D., Hou Z., Lv K., Kan G. (2013). MiR-214 targets ATF4 to inhibit bone formation. Nat. Med..

[43] Zhao C., Sun W., Zhang P., Ling S., Li Y., Zhao D., Peng J., Wang A., Li Q., Song J. (2015). RNA Biology miR-214 promotes osteoclastogenesis by targeting Pten/PI3k/Akt pathway miR-214 promotes osteoclastogenesis by targeting Pten/PI3k/Akt pathway. RNA Biol..

[44] Ikebuchi Y., Aoki S., Honma M., Hayashi M., Sugamori Y., Khan M., Kariya Y., Kato G., Tabata Y., Penninger J.M. (2018). Coupling of bone resorption and formation by RANKL reverse signalling. Nature.

[45] Ge M., Ke R., Cai T., Yang J., Mu X. (2015). Identification and proteomic analysis of osteoblast-derived exosomes. Biochem. Biophys. Res. Commun..

[46] Saito A., Ochiai K., Kondo S., Tsumagari K., Murakami T., Cavener D.R., Imaizumi K. (2011). Endoplasmic reticulum stress response mediated by the PERK-eIF2α-ATF4 pathway is involved in osteoblast differentiation induced by BMP2. J. Biol. Chem..

[47] Zhang P., Mcgrath B., ai Li S., Frank A., Zambito F., Reinert J., Gannon M., Ma K., Mcnaughton K., Cavener D.R. (2002). The PERK Eukaryotic Initiation Factor 2 Kinase Is Required for the Development of the Skeletal System, Postnatal Growth, and the Function and Viability of the Pancreas. Mol. Cell. Biol..

[48] Cui Y., Luan J., Li H., Zhou X., Han J. (2016). Exosomes derived from mineralizing osteoblasts promote ST2 cell osteogenic differentiation by alteration of microRNA expression. FEBS Lett..

[49] Hwang S., Park S.-K., Lee H.Y., Kim S.W., Lee J.S., Choi E.K., You D., Kim C.-S., Suh N. (2014). miR-140-5p suppresses BMP2-mediated osteogenesis in undifferentiated human mesenchymal stem cells. FEBS Lett..

[50] Solberg L.B., Stang E., Brorson S.H., Andersson G., Reinholt F.P. (2014). Tartrate-resistant acid phosphatase (TRAP) co-localizes with receptor activator of NF-KB ligand (RANKL) and osteoprotegerin (OPG) in lysosomal-associated membrane protein 1 (LAMP1)-positive vesicles in rat osteoblasts and osteocytes. Histochem. Cell Biol..

[51] Deng L., Wang Y., Peng Y., Wu Y., Ding Y., Jiang Y., Shen Z., Fu Q. (2015). Osteoblast-derived microvesicles: A novel mechanism for communication between osteoblasts and osteoclasts. Bone.

[52] Chen C., Cheng P., Xie H., Zhou H.-D., Wu X.P., Liao E.Y., Luo X.H. (2014). MiR-503 regulates osteoclastogenesis via targeting RANK. J. Bone Miner. Res..

[53] Sato M., Suzuki T., Kawano M., Tamura M. (2017). Circulating osteocyte-derived exosomes contain miRNAs which are enriched in exosomes from MLO-Y4 cells. Biomed. Rep..

[54] Qin Y., Wang L., Gao Z., Chen G., Zhang C. (2016). Bone marrow stromal/stem cell-derived extracellular vesicles regulate osteoblast activity and differentiation in vitro and promote bone regeneration in vivo. Sci. Rep..

[55] Yang X., Matsuda K., Bialek P., Jacquot S., Masuoka H.C., Schinke T., Li L., Brancorsini S., Sassone-Corsi P., Townes T.M. (2004). ATF4 is a substrate of RSK2 and an essential regulator of osteoblast biology: Implication for Coffin-Lowry syndrome. Cell.

[56] Zhao L., Jiang S., Hantash B.M. (2010). Transforming Growth Factor Β1 Induces Osteogenic Differentiation of Murine Bone Marrow Stromal Cells. Proceedings of the Tissue Engineering—Part A.

[57] Luther G., R. Wagner E., Zhu G., Kang Q., Luo Q., Lamplot J., Bi Y., Luo X., Luo J., Teven C. (2011). BMP-9 Induced Osteogenic Differentiation of Mesenchymal Stem Cells: Molecular Mechanism and Therapeutic Potential. Curr. Gene Ther..

[58] Narayanan R., Huang C.-C., Ravindran S. (2016). Hijacking the Cellular Mail: Exosome Mediated Differentiation of Mesenchymal Stem Cells. Stem Cells Int..

[59] Vallabhaneni K.C., Penfornis P., Dhule S., Guillonneau F., Adams K.V., Mo Y.Y., Xu R., Liu Y., Watabe K., Vemuri M.C. (2015). Extracellular vesicles from bone marrow mesenchymal stem/stromal cells transport tumor regulatory microRNA, proteins, and metabolites. Oncotarget.

[60] Nguyen D.X., Bos P.D., Massagué J. (2009). Metastasis: From dissemination to organ-specific colonization. Nat. Rev. Cancer.

[61] Inder K.L., Ruelcke J.E., Petelin L., Moon H., Choi E., Rae J., Blumenthal A., Hutmacher D., Saunders N.A., Stow J.L. (2014). Cavin-1/PTRF alters prostate cancer cell-derived extracellular vesicle content and internalization to attenuate extracellular vesicle-mediated osteoclastogenesis and osteoblast proliferation. J. Extracell. Vesicles.

[62] Karlsson T., Lundholm M., Widmark A., Persson E. (2016). Tumor cell-derived exosomes from the prostate cancer cell line TRAMP-C1 impair osteoclast formation and differentiation. PLoS ONE.

[63] Itoh T., Ito Y., Ohtsuki Y., Ando M., Tsukamasa Y., Yamada N., Naoe T., Akao Y. (2012). Microvesicles released from hormone-refractory prostate cancer cells facilitate mouse pre-osteoblast differentiation. J. Mol. Histol..

[64] Dai J., Escara-Wilke J., Keller J.M., Jung Y., Taichman R.S., Pienta K.J., Keller E.T. (2019). Primary prostate cancer educates bone stroma through exosomal pyruvate kinase M2 to promote bone metastasis. J. Exp. Med..

[65] Ye Y., Li S.L., Ma Y.Y., Diao Y.J., Yang L., Su M.Q., Li Z., Ji Y., Wang J., Lei L. (2017). Exosomal miR-141-3p regulates osteoblast activity to promote the osteoblastic metastasis of prostate cancer. Oncotarget.

[66] Hashimoto K., Ochi H., Sunamura S., Kosaka N., Mabuchi Y., Fukuda T., Yao K., Kanda H., Ae K., Okawa A. (2018). Cancer-secreted hsa-miR-940 induces an osteoblastic phenotype in the bone metastatic microenvironment via targeting ARHGAP1 and FAM134A. Proc. Natl. Acad. Sci. USA.

[67] Probert C., Dottorini T., Speakman A., Hunt S., Nafee T., Fazeli A., Wood S., Brown J.E., James V. (2019). Communication of prostate cancer cells with bone cells via extracellular vesicle RNA; a potential mechanism of metastasis. Oncogene.

[68] Morhayim J., Van De Peppel J., Demmers J.A.A., Kocer G., Nigg A.L., Van Driel M., Chiba H., Van Leeuwen J.P. (2015). Proteomic signatures of extracellular vesicles secreted by nonmineralizing and mineralizing human osteoblasts and stimulation of tumor cell growth. FASEB J..

[69] Tiedemann K., Sadvakassova G., Mikolajewicz N., Juhas M., Sabirova Z., Tabariès S., Gettemans J., Siegel P.M., Komarova S.V. (2019). Exosomal Release of L-Plastin by Breast Cancer Cells Facilitates Metastatic Bone Osteolysis. Transl. Oncol..

[70] Wen S.W., Sceneay J., Lima L.G., Wong C.S.F., Becker M., Krumeich S., Lobb R.J., Castillo V., Wong K.N., Ellis S. (2016). The biodistribution and immune suppressive effects of breast cancer-derived exosomes. Cancer Res..

[71] Ono M., Kosaka N., Tominaga N., Yoshioka Y., Takeshita F., Takahashi R.U., Yoshida M., Tsuda H., Tamura K., Ochiya T. (2014). Exosomes from bone marrow mesenchymal stem cells contain a microRNA that promotes dormancy in metastatic breast cancer cells. Sci. Signal..

[72] Bliss S.A., Sinha G., Sandiford O.A., Williams L.M., Engelberth D.J., Guiro K., Isenalumhe L.L., Greco S.J., Ayer S., Bryan M. (2016). Mesenchymal stem cell-derived exosomes stimulate cycling quiescence and early breast cancer dormancy in bone marrow. Cancer Res..

[73] Peng X., Guo W., Ren T., Lou Z., Lu X., Zhang S., Lu Q., Sun Y. (2013). Differential Expression of the RANKL/RANK/OPG System Is Associated with Bone Metastasis in Human Non-Small Cell Lung Cancer. PLoS ONE.

[74] Taverna S., Pucci M., Giallombardo M., Di Bella M.A., Santarpia M., Reclusa P., Gil-Bazo I., Rolfo C., Alessandro R. (2017). Amphiregulin contained in NSCLC-exosomes induces osteoclast differentiation through the activation of EGFR pathway. Sci. Rep..

[75] Xu Z., Liu X., Wang H., Li J., Dai L., Li J., Dong C. (2018). Lung adenocarcinoma cell-derived exosomal miR-21 facilitates osteoclastogenesis. Gene.

[76] Valencia K., Luis-Ravelo D., Bovy N., Antón I., Martínez-Canarias S., Zandueta C., Ormazábal C., Struman I., Tabruyn S., Rebmann V. (2014). miRNA cargo within exosome-like vesicle transfer influences metastatic bone colonization. Mol. Oncol..

[77] Yamamoto T., Kosaka N., Ochiya T. (2019). Latest advances in extracellular vesicles: From bench to bedside. Sci. Technol. Adv. Mater..

[78] Vader P., Mol E.A., Pasterkamp G., Schiffelers R.M. (2016). Extracellular vesicles for drug delivery. Adv. Drug Deliv. Rev..

[79] Alvarez-Erviti L., Seow Y., Yin H., Betts C., Lakhal S., Wood M.J.A. (2011). Delivery of siRNA to the mouse brain by systemic injection of targeted exosomes. Nat. Biotechnol..

[80] Sun D., Zhuang X., Zhang S., Deng Z.B., Grizzle W., Miller D., Zhang H.G. (2013). Exosomes are endogenous nanoparticles that can deliver biological information between cells. Adv. Drug Deliv. Rev..

[81] Van Den Boorn J.G., Schlee M., Coch C., Hartmann G. (2011). SiRNA delivery with exosome nanoparticles. Nat. Biotechnol..

[82] Tian Y., Li S., Song J., Ji T., Zhu M., Anderson G.J., Wei J., Nie G. (2014). A doxorubicin delivery platform using engineered natural membrane vesicle exosomes for targeted tumor therapy. Biomaterials.

[83] Ohno S.I., Takanashi M., Sudo K., Ueda S., Ishikawa A., Matsuyama N., Fujita K., Mizutani T., Ohgi T., Ochiya T. (2013). Systemically injected exosomes targeted to EGFR deliver antitumor microrna to breast cancer cells. Mol. Ther..

[84] Manca S., Upadhyaya B., Mutai E., Desaulniers A.T., Cederberg R.A., White B.R., Zempleni J. (2018). Milk exosomes are bioavailable and distinct microRNA cargos have unique tissue distribution patterns. Sci. Rep..

[85] Somiya M., Yoshioka Y., Ochiya T. (2018). Biocompatibility of highly purified bovine milk-derived extracellular vesicles. J. Extracell. Vesicles.

[86] Trajkovic K., Hsu C., Chiantia S., Rajendran L., Wenzel D., Wieland F., Schwille P., Brügger B., Simons M. (2008). Ceramide triggers budding of exosome vesicles into multivesicular endosomes. Science.

[87] Essandoh K., Yang L., Wang X., Huang W., Qin D., Hao J., Wang Y., Zingarelli B., Peng T., Fan G.C. (2015). Blockade of exosome generation with GW4869 dampens the sepsis-induced inflammation and cardiac dysfunction. Biochim. Biophys. Acta Mol. Basis Dis..

[88] Marleau A.M., Chen C.S., Joyce J.A., Tullis R.H. (2012). Exosome removal as a therapeutic adjuvant in cancer. J. Transl. Med..

[89] Ciravolo V., Huber V., Ghedini G.C., Venturelli E., Bianchi F., Campiglio M., Morelli D., Villa A., Mina P.D., Menard S. (2012). Potential role of HER2-overexpressing exosomes in countering trastuzumab-based therapy. J. Cell. Physiol..

[90] Nishida-Aoki N., Tominaga N., Takeshita F., Sonoda H., Yoshioka Y., Ochiya T. (2017). Disruption of Circulating Extracellular Vesicles as a Novel Therapeutic Strategy against Cancer Metastasis. Mol. Ther..

[91] Mulcahy L.A., Pink R.C., Carter D.R.F. (2014). Routes and mechanisms of extracellular vesicle uptake. J. Extracell. Vesicles.

[92] Lipton A., Zheng M., Seaman J. (2003). Zoledronic acid delays the onset of skeletal-related events and progression of skeletal disease in patients with advanced renal cell carcinoma. Cancer.

[93] Smith M.R., Saad F., Coleman R., Shore N., Fizazi K., Tombal B., Miller K., Sieber P., Karsh L., Damião R. (2012). Denosumab and bone-metastasis-free survival in men with castration-resistant prostate cancer: Results of a phase 3, randomised, placebo-controlled trial. Lancet.

[94] Henry D.H., Costa L., Goldwasser F., Hirsh V., Hungria V., Prausova J., Scagliotti G.V., Sleeboom H., Spencer A., Vadhan-Raj S. (2011). Randomized, Double-Blind Study of Denosumab Versus Zoledronic Acid in the Treatment of Bone Metastases in Patients With Advanced Cancer (Excluding Breast and Prostate Cancer) or Multiple Myeloma. J. Clin. Oncol..

